# Psychometric properties and detectability of GPT-4o–generated multiple-choice questions compared with human-authored items across imaging specialties

**DOI:** 10.1038/s41746-025-02313-7

**Published:** 2026-01-08

**Authors:** Philipp Linde, Florian Fichter, Markus Dietlein, Ferdinand Sudbrock, Kambiz Afshar, Hendrik Dapper, Emmanouil Fokas, Anna-Lena Hillebrecht, Tobias Raupach, Matthias Carl Laupichler

**Affiliations:** 1 Department of Radiation Oncology, Cyberknife and Radiation Therapy, Faculty of Medicine and University Hospital of Cologne, University of Cologne, Cologne, Germany; 2 Institute for Diagnostic and Interventional Radiology, Faculty of Medicine and University Hospital Cologne, University of Cologne, Cologne, Germany; 3 Department of Nuclear Medicine, Faculty of Medicine and University Hospital of Cologne, University of Cologne, Cologne, Germany; 4 Institute for General Practice and Palliative Care, Hannover Medical School, Hannover, Germany; 5 Department of Prosthetic Dentistry, Centre for Dental Medicine, Medical Centre, University of Freiburg, Freiburg, Germany; 6 Institute of Medical Education, University Hospital Bonn, Bonn, Germany

**Keywords:** Cancer, Health care, Medical research, Oncology

## Abstract

Large language models (LLMs) have the potential to scale assessment in medical education, but their psychometric equivalence to expert-written items and the detectability of their origin remain uncertain. In a preregistered, single-center, blinded observational, within-subject comparison, we evaluated 24 GPT-4o–generated versus 24 human-authored topic-matched multiple-choice questions (MCQs) across radiation oncology, radiology, and nuclear medicine. Medical students (*n* = 82) and physicians (*n* = 46) completed an identical 48-item formative mock examination, with item origin masked. Item difficulty (human: mean 0.65 [SD 0.22] vs LLM: 0.67 [0.20]) and discrimination (0.27 [0.12] vs 0.29 [0.12]) did not differ significantly; participants did not identify item origin above chance (0.50). Expert ratings of appropriateness and didactic quality showed low interrater agreement (ICC = 0.07–0.18). In this expert-reviewed, human-in-the-loop workflow, the item difficulty and discriminatory power of MCQs generated with GPT-4o did not differ significantly from those of expert-authored items, and were not reliably recognized as AI-generated by examinees. These findings delineate a feasible pathway for responsibly scaling formative assessment content in imaging-focused medical education, while underscoring the need for explicit educational policies regarding oversight, transparency, and fairness.

## Introduction

Large language models (LLMs) have rapidly evolved from technological curiosity to foundational infrastructure across medicine, with fast-growing evidence in clinical analytics, imaging, and decision support^1–4^. Domain-adapted foundation models are already being deployed in radiology-centric tasks and oncology workflows^5,6^, underscoring how quickly artificial intelligence (AI) capabilities are converging on specialty needs and multimodal use cases. In parallel, educational practice is changing just as rapidly: trainees are adopting generative AI at scale, yet proficiency and trust vary widely^7^, highlighting a gap between real-world use and evidence-based guidance on when—and how—AI should enter teaching and assessment^8^.

Building and maintaining high-quality multiple-choice question (MCQ) banks demands subject-matter expertise^9^, sustained faculty effort, and psychometric vetting^10^.

These pressures are well recognized in medical education and across clinical disciplines. On the one hand, the extensive literature on the testing effect^11^ and test-enhanced learning^12^ demonstrates that frequent formative assessment with high-quality test items significantly improves the learning outcomes of medical students. Accordingly, it is essential that medical students have access to a broad set of practice questions. On the other hand, rapidly evolving evidence, guideline updates, and increasing subspecialty fragmentation create an ongoing need for new, reliable items.

LLMs offer the potential to accelerate first-draft authoring^13^, but two questions remain central to determining their reliability and validity: (i) Do AI-generated items match expert-written items on core psychometric properties (difficulty, discrimination)? and (ii) Can examinees or experts reliably recognize AI authorship, with implications for integrity and acceptance? Emerging evidence from adjacent domains suggests that modern models can emulate expert output to a degree that eludes naive detection^14^, but rigorous, domain-specific validation with learners and clinicians remains limited.

Meanwhile, the broader AI literature in healthcare disciplines underscores why such validation is essential^2^: specialty-tuned LLMs trained on clinical narratives can achieve high discriminative performance and generalize across institutions^15^, yet they also reveal the importance of transparency, versioning, and human oversight to mitigate domain drift and error propagation. Foundation models for medical imaging demonstrate how scaling and promptability can unlock breadth^16^, but educational policies and curricular governance^17^—through clear task definitions, expert review, and post-hoc performance monitoring—ultimately determines safe translation^10^. Recent evaluations of LLMs in medical education across diverse clinical and cultural contexts similarly emphasize both opportunity and risk^18^, and advocate for study designs that blind raters to item origin and benchmark AI outputs against expert standards^19^.

Previous studies examining LLM-generated assessment items in undergraduate medical education have provided initial evidence that generative AI is at least partially suitable for producing formative assessments^20^. However, these studies evaluated question quality either solely through expert review^21,22^, through comparison with guidelines, or focused on other item formats such as key-feature questions or script concordance tests^23–25^. An early literature review by Artsi et al.^26^ emphasized that the quality of study designs in this area still requires improvement. More recently, a growing number of studies have reported results suggesting that LLM-generated MCQs exhibit item parameters comparable to those of human-created items. Nonetheless, in many cases, a substantial proportion of AI-generated MCQs must be excluded due to errors or unclear wording^27–29^.

While this study addresses the research questions through a blinded, within-subject observational comparison across three imaging specialties (radiation oncology, radiology, and nuclear medicine), the methodological approach and its implications for item development are broadly transferable to other clinical disciplines. GPT-4o–generated and human-authored MCQs on matched topics were administered to medical students and clinical physicians. The primary metrics are item difficulty (the proportion of examinees answering an item correctly, with higher values indicating easier items) and discriminatory power (the extent to which an item differentiates higher- from lower-performing examinees). Secondary outcomes include participants’ ability to identify item origin and expert ratings of appropriateness and didactic quality, with interrater reliability quantified. A human-in-the-loop process screens AI-generated items for factual accuracy prior to administration. Informed by multicenter evidence that trainee adoption is widespread but confidence and competency remain heterogeneous^15^, we examine whether LLM-generated items can augment assessment while preserving psychometric quality and functional indistinguishability.

Therefore, we prespecified three questions: (1) whether AI-generated items would show no statistically significant differences from expert-authored items on item difficulty and discrimination; (2) whether examinees and experts would identify item origin no better than chance (0.50); and (3, exploratory) whether clinicians in imaging disciplines differ from medical students in origin identification. Accordingly, we hypothesized that there is no statistically significant difference in core psychometric indices (item difficulty and discriminatory power) between GPT-4o–generated items and expert items (hypothesis (H) 1), that origin identification would not be significantly different from chance (H2), and that there would be no significant differences regarding psychometric indices or item origin identification between both groups (clinicians vs students) (H3).

Accordingly, we conducted a preregistered, blinded, within-subjects study across three imaging specialties, comparing GPT-4o–generated with human-written multiple-choice questions on item difficulty, point-biserial discrimination, and internal consistency; with provenance detectability as a secondary outcome.

## Results

### Participant characteristics

A total of [*N* = 128] participants completed the study, including [n_s_ = 82] medical students and [n_p_ = 46] physicians. All participants received the same 48-item questionnaire (mock examination) (2 × 24 human vs LLM; origin masked). Among physicians, [*n* = 20 (43%)] were board-certified and [*n* = 24 (52%)] in specialty training; primary disciplines were radiation oncology ([*n* = 15 (33%)]), nuclear medicine ([*n* = 7 (15%)]), and radiology ([*n* = 5 (11%)]), with ([*n* = 18 (39%)]) coming from other disciplines (e.g., internal medicine).

Missingness was low (≤[4.7]% across variables). Analyses used complete-case data; no imputation was conducted. Unanswered items were coded as incorrect and therefore not treated as missing in psychometric analyses. No demographic variables (age, sex/gender) were collected. Full sample characteristics are presented in Table 1.

**Table 1:** Participant characteristics by qualification and specialty (*N* = 128)

	Absolute (n)	Relative (%)
**Medical students**	82	64.1
**Physicians**	46	35.9
In training (residents)	24	52.2
Board-certified	20	43.5
Not disclosed	2	4.3
**Primary discipline (physicians)**		
Radiation oncology	15	32.6
Radiology	5	10.9
Nuclear medicine	7	15.2
Other (e.g., internal medicine)	18	39.1
Not disclosed	1	2.2

*Note*. “Not disclosed” indicates the variable was left blank.

### Item parameters of human- and LLM-generated questions (H1)

**Fig. 1:**
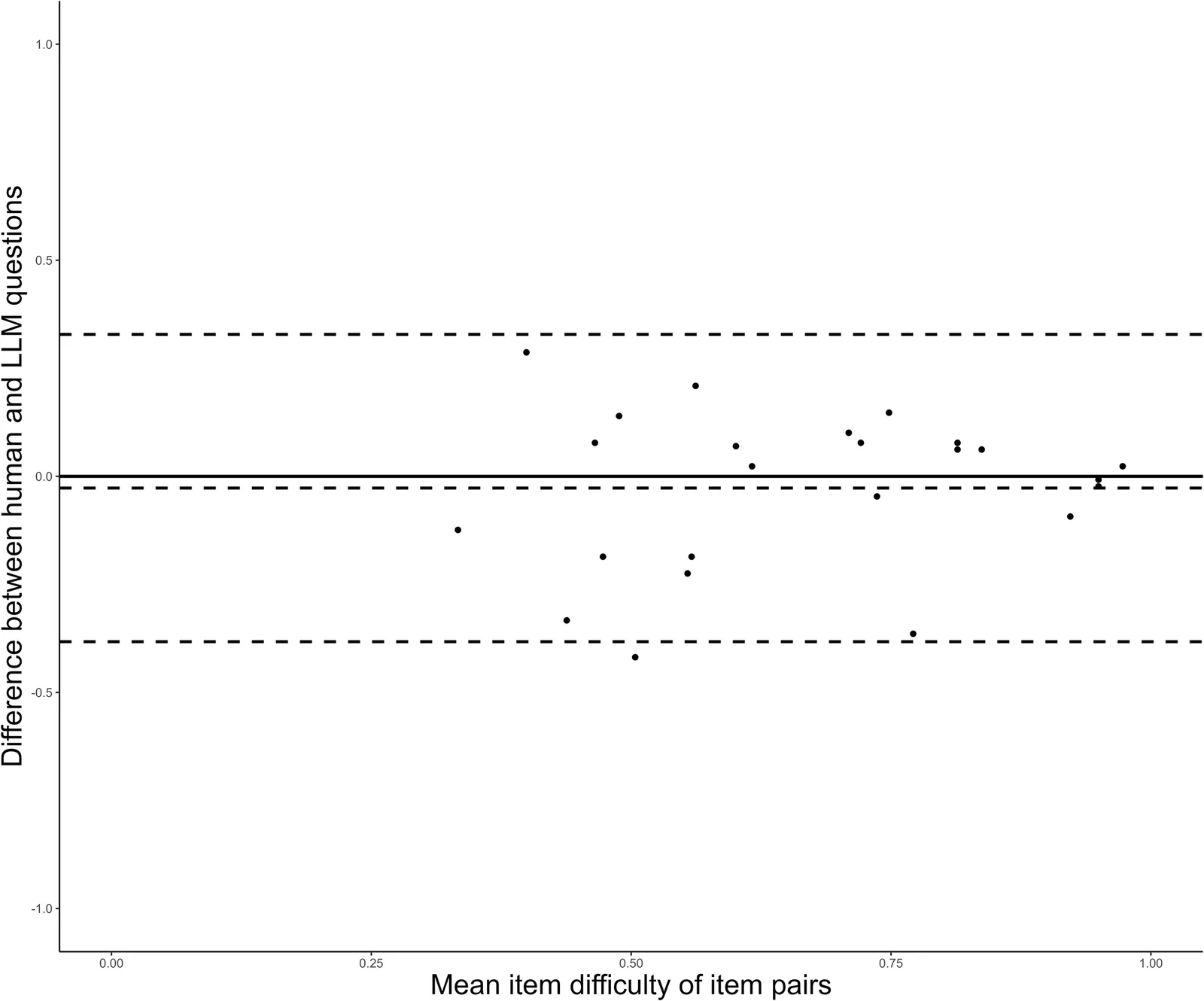
I Bland-Altman plot illustrating the difference in item difficulty between pairs of human-authored and LLM-generated questions on the same topic. *Note*. Bland-Altman plot depicting the differences in item difficulty between exam questions developed by human and LLM-generated questions, University of Cologne Medical School, summer 2025. There were 24 item pairs of human and LLM questions. Each dot represents the difference in item difficulty between the human-authored and LLM-generated multiple-choice question on the same topic that was used in a preparatory exam. The central dotted line indicates the mean difference between human and LLM questions, while the outer dashed lines denote the upper and lower limit of agreement (i.e., the upper and lower end of the 95% confidence interval). LLM questions were generated by ChatGPT 4o (April 25, 2025, version; OpenAI, San Francisco, CA). Abbreviation: LLM, large language model. The figure was generated using R (version 4.2.2; R Core Team, 2023) and RStudio (version 2024.12.0 + 467; Posit Team, 2024).

During the human-in-the-loop curation process, 2 of the 24 topics (8%) required re-prompting because both initial GPT-4o-generated candidates were judged unsuitable according to our predefined criteria (one radiation oncology topic (STH-Q3, 10 May 2025), and one radiology topic (RAD-Q4, 15 May 2025)). Regarding the hypothesis, item difficulty did not differ significantly between human-authored (*M* = 0.65, *SD* = 0.22) and LLM-generated questions (*M* = 0.67, *SD* = 0.20) in the total sample, *t*(45.52) = –0.446, *p* = 0.658 (see Fig. 1).

**Fig. 2:**
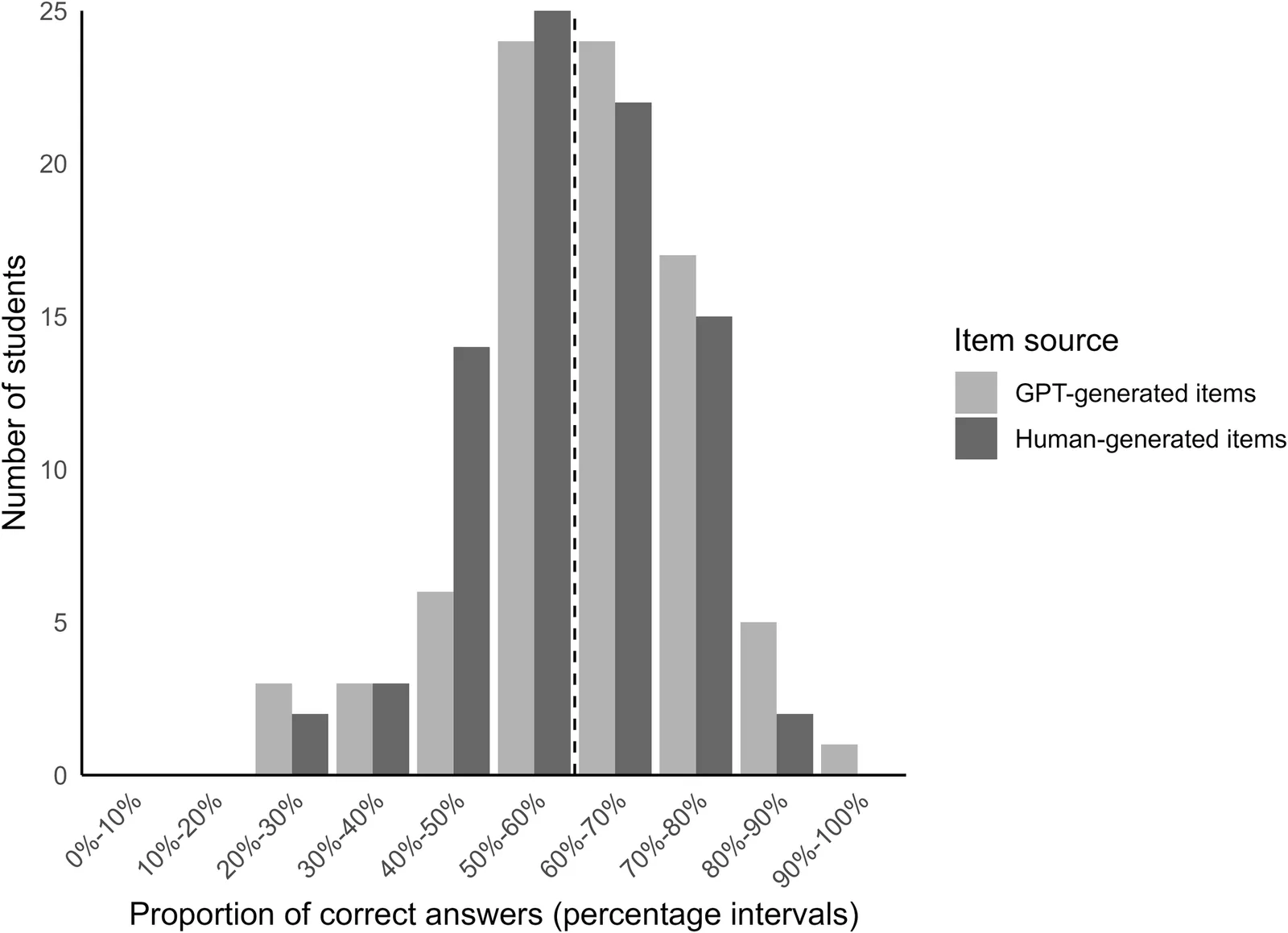
I Bar chart comparing medical students’ performance on human-authored and LLM-generated questions. *Note*. Bar chart comparing medical students’ [n_s_ = 82] performance on human-authored and LLM-generated questions, University of Cologne Medical School, summer 2025. Each student’s percentage score was analyzed separately for the 24 human-authored and 24 LLM-generated items. The percentage values were grouped into corresponding brackets (e.g., 30–40%). The height of each bar represents the number of students whose performance fell within the respective bracket. Light gray bars indicate performance on LLM-generated questions, while dark gray bars indicate performance on human-authored questions. The dashed line indicates the threshold that must be exceeded to pass the examination in many medical schools. The figure was generated using R (version 4.2.2; R Core Team, 2023) and RStudio (version 2024.12.0 + 467; Posit Team, 2024).

Similarly, no significant difference in item difficulty was observed in the medical student subsample (human-authored: *Mdn* = 0.58, *SD* = 0.26; LLM-generated: *Mdn* = 0.59, *SD* = 0.22), *z* (N_1_ = 24, N_2_ = 24) = –0.413, *p* = 0.680, nor in the physician subsample (human-authored: *M* = 0.76, *SD* = 0.17; LLM-generated: *M* = 0.78, *SD* = 0.18), *t*(45.94) = –0.399, *p* = 0.692. Because percentage performance in examinations determines whether students pass or fail in many medical schools, Fig. 2 presents the percentage distribution of medical students’ performance on human-authored and LLM-generated questions (see Fig. 2).

Likewise, discriminatory power did not differ significantly between human-authored (*M* = 0.27, *SD* = 0.12) and LLM-generated questions (*M* = 0.29, *SD* = 0.12) in the total sample, *t*(45.87) = –0.425, *p* = 0.673. This finding also held in the medical student subsample (human-authored: *M* = 0.18, *SD* = 0.12; LLM-generated: *M* = 0.20, *SD* = 0.15), *t*(44.40) = –0.680, *p* = 0.500, as well as in the physician subsample (human-authored: *M* = 0.24, *SD* = 0.19; LLM-generated: *M* = 0.24, *SD* = 0.20), *t*(45.88) = –0.049, *p* = 0.961.

### Ability to distinguish human- and LLM-generated questions (H2)

Participants’ ability to correctly identify the origin of the multiple-choice questions (*M* = 0.49, *SD* = 0.12) did not differ significantly from chance level (0.50), *t*(47) = -0.787, *p* = 0.435. A similar pattern was observed in the medical student subsample (non-normally distributed; *Mdn* = 0.51, *SD* = 0.13), *Z* = -0.28, *p* = 0.778, as well as in the physician subsample (*M* = 0.49, *SD* = 0.12), *t*(47) = -0.606, *p* = 0.548.

### Differences between students’ and physicians’ ability to distinguish question source (H3)

In regard of the third hypothesis, medical students (Mdn = 0.51, SD = 0.13) and physicians (Mdn = 0.51, SD = 0.13) did not differ in their ability to distinguish between human-authored and LLM-generated questions, *z* (N_1_ = 48, N_2_ = 48) = –0.033, *p* = 0.974.

### Expert evaluation of item content

A single fixed-rater intraclass correlation analysis for the evaluation of the appropriateness of questions for use in undergraduate medical examinations, based on ratings from all four experts, yielded an ICC of 0.18, *F*(23, 69) = 1.8, *p* = 0.026. According to established criteria^30^ and more recent guidelines, this represents a low level of interrater reliability. A similar pattern emerged for the evaluation of didactic quality, with an ICC of 0.07, *F*(23, 69) = 1.3, *p* = 0.200, also indicating weak agreement.

When separating the ratings into two groups – clinical experts and medical education experts – most Spearman rank-order correlations between rater pairs were not statistically significant. An exception was found for the evaluation of appropriateness by the medical education experts, whose ratings showed a strong and statistically significant correlation, ρ = 0.543, *p* < 0.01.

Interrater agreement regarding the identification of item origin was also very low, with Fleiss’ Kappa κ = –0.015.

## Discussion

In this single-center, blinded within-subject experiment using 48 imaging-focused MCQs across radiology, nuclear medicine, and radiation oncology, GPT-4o–generated items showed no statistically significant differences in item difficulty or discrimination compared with human-authored items, and examinees could not identify item origin above chance level. Embedded within a human-in-the-loop framework with structured expert oversight, this study evaluated whether generative models could responsibly augment assessment in medical education while maintaining psychometric quality and aiming to safeguard fairness and trust. The findings contribute to the growing discourse on responsible AI integration in medical imaging education by addressing the dual imperative of innovation and educational policy. Since assessment drives learning, it is essential to determine whether LLMs can safely augment, rather than replace, item development under human oversight. Our blinded, within-subject evaluation suggests that GPT-4o–generated questions can match expert-written items on core psychometric properties in a formative mock examination across imaging disciplines.

1. Analysis revealed that GPT-4o–generated questions achieved psychometric parity with expert-written questions: there were no significant differences in item difficulty or discrimination. In this focused, imaging-oriented setting, a state-of-the-art LLM was able to emulate the technical performance of human-crafted items. This indicates that generative AI could feasibly help expand radiation oncology, radiology, and nuclear medicine question banks while preserving core psychometric properties in formative assessment contexts, provided structured human oversight is in place.

This is notable given the persistent need for new and up-to-date questions in medical education^31–33^. At the same time, we emphasize that similar psychometric indices do not imply that AI can replace human educators. Rather, it indicates AI augmentation is feasible when paired with appropriate oversight^34,35^.

(2) Equally important, participants could not distinguish AI-generated items from human-written items above chance level. Neither medical students nor experienced clinicians in our study were reliably able to tell whether a given question was authored by GPT-4o or by a human expert. The inability of typical end users to detect item provenance is educationally relevant in itself, because these are the groups that would ultimately consume such questions in formative and, potentially, high-stakes assessment. The inability to identify item origin aligns with prior work^27^ and suggests that the GPT-4o questions were not reliably distinguishable by examinees in this setting in style and substance from conventional questions. From an exam integrity standpoint, the result is encouraging: it implies that LLM-generated questions can be potentially integrated into formative assessments without tipping off or distracting examinees.
(3) Consistent with rater-dependent variability, interrater reliability among four experts for rating question appropriateness and didactic quality was limited (ICC values in the 0.1–0.2 range). The ICC was computed on a subset of 24 items, and based on only four raters, which may have reduced precision and attenuated the coefficient, and may indicate minimal agreement on the merits of individual items. This outcome underscores that even experienced educators and clinicians may disagree on what constitutes a “good” question. The divergence in expert ratings suggests that both AI-generated and human-written items were acceptable to some raters—and occasionally preferred—while others judged them less favorably. Consistent with prior work documenting rater-dependent variability and frequent item-writing flaws^36–38^, this pattern supports multi-expert review with calibrated rubrics and explicit reliability checks (e.g., ICC) prior to deployment, regardless of origin, as different perspectives (didactical vs. clinical) can yield to different appraisals of an item’s value^39,40^. The expert ratings should not be interpreted as definitive evidence that human-authored items are didactically superior—or vice versa—but rather as highlighting that even experienced clinicians and medical educators may differ markedly in their perceptions of item quality. The implication is that multi-expert review processes should explicitly monitor and, where possible, improve interrater reliability through calibrated rating rubrics, consensus discussions, or additional training, irrespective of whether items originate from human authors or LLMs.

Our approach incorporated a human-in-the-loop review that identified and corrected infrequent factual errors in the AI-generated questions. Despite GPT-4o’s advanced capabilities, a very small number of the generated items contained inaccuracies or outdated information. We removed or edited these before the questions were used in the study’s exam. This step was critical: even a single unchecked error in an examination question can confuse learners and undermine the test’s credibility^41^. In essence, our results endorse a model of AI–human collaboration: the LLM serves as a capable first draft generator, and human experts serve as editors and final reviewers of quality. In practice, this human-in-the-loop approach is not only about factual accuracy; it is also about aligning items with curricular relevance, cognitive level, and institutional standards of fairness^42^.

In educational assessment, fairness also extends the content of questions: language, context, and underlying assumptions should not introduce bias^43,44^. In this study, GPT-4o was prompted to create questions on specified medical topics, which likely constrained it to professional, bias-neutral phrasing. Indeed, no participant or expert feedback indicated any offensive or biased content. Nonetheless, as generative models draw on vast internet-trained data, ongoing monitoring for hidden biases is warranted^45,46^. Subtle biases in clinical scenarios or word choice could emerge if an AI’s training data contained skewed representations. Institutions should consider transparency around AI use^47^ to maintain trust: fairness is not only a conceptual quality criterion but also a perception. If students are aware that some questions are AI-generated, they may question whether these items were subject to equivalent scrutiny. Clearly communicating (when appropriate) that all exam items, regardless of origin, underwent the same rigorous review could help preserve confidence in the assessment’s fairness.

This study has several limitations that temper our findings. First, it was conducted at a single medical school, with a limited number of participants (particularly among clinicians) and using only 48 questions across three subdomains in a formative mock examination. These factors limit generalizability rather than internal validity, despite adequate power for the primary endpoints. In other medical specialties, with different types of questions or a more diverse examinee cohort, the performance of AI-generated items could diverge from what we observed. Despite blinding and the within-subjects design, residual selection bias is possible due to the single-center, volunteer sample; order effects cannot be fully excluded; and investigators could not be blinded during item generation and pre-vetting. Second, we focused on one specific LLM (OpenAI’s GPT-4o) even if the study was not designed as an inter-model benchmark. LLM technology is evolving rapidly; newer models or updates may perform differently, and other available models might not reach GPT-4o’s level of quality. Caution is therefore warranted in extrapolating our findings; future work should extend this protocol to multiple models and versions to assess generalizability across systems and update cycles. Third, although our analysis compared major psychometric properties and detection rates, there are other dimensions of question quality we did not explicitly measure: higher-order cognitive complexity, integration of visual information (for imaging questions), and alignment with curriculum standards are all critical for good examination items. Our AI-generated questions were similar to human ones in difficulty and discrimination, but we did not evaluate whether they were equally effective at testing application of knowledge vs. recall. Subtle differences might exist in how AI-crafted questions are written—perhaps in phrasing or distractor quality—that were not captured by our metrics but could impact learning outcomes. Because multiple items per specialty were generated within a single chat session, subtle within-thread stylistic carryover remains possible; we did not perform a formal similarity analysis. We also did not formally classify item cognitive level (e.g., using Bloom’s taxonomy). Consequently, we cannot determine whether AI-generated and human-written items differed systematically in cognitive demand. Future studies should use prespecified coding frameworks and have multiple independent raters with inter-rater agreement analysis, and should generate each AI item in an independent session with lexical similarity metrics to quantify overlap. Importantly, item difficulty values (proportion correct) were broadly within acceptable ranges for both GPT-4o–generated and human-written items. No item fell into an implausibly low difficulty range (<0.20 for a five-option MCQ), and only six items were below 0.40, four of which were human-written and two AI-generated. Conversely, very easy items (>0.80 correct) occurred in both groups. This pattern suggests that AI-generated questions were neither systematically “too hard” nor “too easy” compared with expert-written questions, at least in our formative setting. We therefore did not observe evidence that GPT-4o–generated items would disadvantage examinees in terms of overall test performance; however, this inference is limited to a formative assessment context and may not directly translate to high-stakes summative examinations. Although the within-subject design allowed item responses to be compared within the same individuals, it also had potential disadvantages: participants might theoretically infer the correct answer to one MCQ from a parallel MCQ on the same topic but of a different origin. Relatedly, our expert review, despite catching overt errors, might have missed more nuanced issues (such as slightly ambiguous wording or less optimal distractors in some AI-generated items). The expert panel combined clinical and pedagogical viewpoints rather than to mirror the full spectrum of imaging subspecialties. While this diversity supports general judgments of appropriateness and didactic quality, the absence of dedicated radiology and nuclear medicine specialists may have reduced sensitivity for discipline-specific evaluation, including for origin judgments. We therefore interpret the expert ratings as broad indicators of perceived quality across specialties and explicitly acknowledge panel composition as a limitation rather than as a definitive specialty-specific assessment. We did not assess inter-operator variability; future work should compare outputs when multiple educators execute the same standardized prompt independently. Finally, we did not investigate how awareness of AI-generated content might influence examinees perceptions or acceptance, an important consideration for future implementation.

To our knowledge, this is the first study to compare GPT-4o–generated with human-written multiple-choice questions across imaging subspecialties using a within-subjects design with medical students and clinicians. The results have implications for curriculum design – particularly in radiation oncology, radiology, and nuclear medicine–and for assessment strategy in the age of AI. By reducing the time required to draft new questions, LLMs may enable more frequent formative assessments and a broader coverage of curricular topics. Educators might leverage GPT-4o to generate question variants on emerging medical knowledge, such as new imaging techniques or updated guidelines, helping examinations and study materials remain current. Beyond single best-answer MCQs, LLMs could also be used to draft key-feature cases that test procedural knowledge and clinical decision-making, allowing educators to iteratively refine complex, scenario-based items rather than writing them from scratch. This agility in refreshing and expanding item banks aligns with the needs of dynamic fields like ours, where staying current is a challenge for traditional item development cycles.

Based on our experience, we outline four practical checkpoints for responsible integration of AI-generated items: (i) prompt generation anchored to defined learning objectives; (ii) expert review by medical education faculty to correct factual errors, flag ambiguity, and screen for bias; (iii) blinded pilot testing with psychometric screening; and (iv) institutional decisions about appropriate use cases and disclosure of AI authorship. We view these as core elements of educational governance and digital professionalism. As AI becomes increasingly embedded in medical education, responsible integration will depend on faculty who can supervise and audit AI output, and on learners who understand both its strengths and its limitations.

Ultimately, our study illustrates both the promise and the boundaries of LLM-assisted assessment design: while AI can generate psychometrically robust items under supervision, human oversight, transparency, and fairness safeguards remain indispensable. AI-assisted question generation should therefore be seen not as a replacement for educational judgment, but as a structured collaboration between clinicians, medical educators, and intelligent systems.

## Methods

### Study design

This study was designed as an exploratory, comparative single-center observational trial and was conducted at a large German medical school (University of Cologne) during the summer term of 2025. The primary objective was to psychometrically validate examination questions generated by a large language model (LLM) against those written by human experts. The validation followed a within-subjects approach at the item level: each participant answered a combination of human- and LLM-generated questions, enabling paired comparisons of item parameters.

Blinding of item provenance (human vs LLM) was implemented to minimize expectation/performance bias and potential algorithm aversion or anti-AI bias, as well as detection bias in provenance judgments. The within-subjects comparison controls for between-participant confounding (i.e., differences in baseline ability or test-taking style) and reduces allocation bias, since every participant contributes data to both item sources.

We intentionally used a single, widely available LLM (ChatGPT; GPT-4o-Plus, model release 25 April 2025) to isolate the effect of AI authorship under human-in-the-loop governance rather than to benchmark multiple models. This choice (i) reflects the tool most commonly accessible to learners and faculty in routine educational settings, (ii) enables version-specific reproducibility of outputs and prompts, and (iii) avoids confounding from model-to-model variability and prompt retuning.

All participants provided written informed consent prior to data collection. No personal identifying information was collected, in line with the study’s focus on item-level analysis and anonymity of responses. The only grouping variables were participant type (student or physician) and, for physicians, their specialty and training status. The study’s primary endpoints were classical item metrics—difficulty and discrimination—for human-authored versus AI-generated questions, while secondary endpoints included participants’ ability to identify an item’s origin and expert ratings of item quality.

To mitigate the risk of a low participation, the study was advertised both directly in teaching sessions, tumor boards, and through social media, mailing lists, posters, and support from the student representatives. All participants were offered individualized performance feedback and access to the complete set of practice questions after the examination.

Data were collected using paper-and-pencil questionnaires. Ultimately, only data from participants who provided consent and completed the exam were analyzed. Participants did not receive any financial incentive.

### Ethical approval, preregistration, and reporting guidelines

The study protocol was approved by the local institutional ethics committee (approval no. 25-1043). Procedures involving human participants were conducted in accordance with the ethical standards of the institutional research committee and the 1964 Helsinki Declaration and its later amendments or comparable ethical standards^48^.

The study protocol and statistical analysis plan were preregistered on the Open Science Framework (OSF; https://osf.io/d67fy) prior to data collection. Reporting followed STROBE (Strengthening the Reporting of Observational Studies in Epidemiology)^49^ recommendations for observational studies. Clinical trial number: not applicable.

### Participants

The student sample was drawn from an optional exam-preparation course for fourth-year medical students. Recruitment took place during a mock exam session in the “Querschnittsbereich 11” (cross-sectional module 11, encompassing imaging disciplines) of the clinical curriculum. The mock exam was scheduled approximately 40 days before the actual final examination to minimize over-preparation and ceiling effects. All students enrolled in the course were invited to participate in the study voluntarily, with the assurance that non-participation would carry no academic disadvantages. At the start of the session, students received written information about the study and provided written informed consent before participation.

In parallel, practitioners from the Departments of Radiology, Nuclear Medicine, Radiation Oncology at the same institution, as well as interested colleagues from related departments, were invited to participate, providing a heterogeneous and representative sample of imaging-related and general clinical backgrounds. This physician group included both board-certified specialists and residents in training (fellows or registrars).

While the physicians, unlike the students, did not take the exam in a lecture hall, efforts were made to ensure that they completed the exam under the same conditions as the students. This was achieved by providing the physicians with the same paper-based exams and the same amount of time to answer the questions. In addition, the physicians completed the exam in equivalent proctored, isolated settings, away from clinical duties. The use of any aids was likewise not permitted.

Participants were eligible if they were adults (≥18 years) and either (a) medical students in the clinical phase of training (fourth year) or (b) practicing physicians (including residents). There were no additional eligibility criteria beyond these requirements and provision of informed consent. Individuals who had been involved in the expert review of item content were not eligible to participate as test-takers.

An a priori power analysis was conducted to determine the minimum number of participants required for adequate statistical power. Assuming a medium effect size (Cohen’s d ≈ 0.5), a two-tailed α = 0.05, and power (1–β) = 0.95 for detecting differences in item metrics between human and AI-generated questions, we targeted a minimum of 34 participants in each group. This calculation was based on a paired t-test (each participant encountering both item types) and indicated that about 64 total participants (34 students and 34 physicians) would be sufficient to detect a 0.5 standard deviation difference in item performance. Given that over 200 students were enrolled in the course and a pool of eligible physicians was available, achieving this sample size was considered feasible.

### Item development

A total of 24 question topics were selected to represent key concepts across the three imaging specialties (8 per specialty). Topic selection was aligned with the German National Competence-Based Learning Objectives Catalog for Medicine^50^, the institutional imaging curriculum, and standard specialty textbooks and lecture materials in radiology, nuclear medicine, and radiation oncology. The items targeted core concepts appropriate for fourth-year medical students.

For each topic, one multiple-choice question (MCQ) was authored by a human expert. Experts were board-certified specialists in radiology, radiation oncology, or nuclear medicine with several years of clinical practice and formal experience in medical assessment and exam item writing. No single individual authored all human-written questions. The corresponding learning objective and item specification were then extracted and used to generate a matching MCQ with GPT-4o via a standardized prompt, resulting in 24 topic-matched pairs (48 items in total).

All questions were formulated in a single-best-answer format: each item consisted of a vignette or question stem and a list of five answer options with one correct answer and several plausible distractors designed to avoid obvious cues. The items were designed to match the cognitive demands of fourth-year imaging assessments, emphasizing structured recall and application of knowledge in short clinical scenarios rather than rote memorization or advanced subspecialty reasoning.

To ensure a fair comparison, all items were newly created for this study – none were taken or adapted from existing question banks or prior examinations. The human-authored questions were cross-checked against the local assessment repository to avoid duplicates or near-duplicates of existing examination items. This approach prevented participants from recognizing questions based on past exposure, which could have confounded judgment about item origin. All items were written in German, corresponding to the language of instruction and examination at the institution.

Subject matter experts (*n* = 8) from the departments of radiation oncology, radiology, and nuclear medicine drafted MCQs for topics within their respective specialties. Every human-generated question then underwent independent peer review by a second expert to verify medical accuracy, clarity of wording, and didactic quality of the item. Questions were edited as needed to resolve any ambiguities or errors before finalization. Through this iterative process, we ensured that the set of expert-written questions was internally consistent and of uniformly high quality across all specialties.

The AI-derived items were created using the large language model GPT-4o-Plus (OpenAI, San Francisco, CA, using the ChatGPT interface, version release April 25, 2025). The standardized prompt used to generate LLM items was co-developed by two authors (PL, MCL) with expertise in medical education and psychometrics. To ensure procedural consistency and reduce operator-induced variability, a single operator (PL; single ChatGPT account) executed all prompts. A board-certified specialist from the relevant discipline attended each prompting session to confirm topical fidelity. For each imaging specialty and for each respective learning objective and specification, the model was prompted to generate a high-quality multiple-choice question on the respective topic, targeting a difficulty level similar to that of the human-authored item. The LLM was not fine-tuned for this study; all outputs were generated by the off-the-shelf model using its pretrained parameters at the time of data collection. Prior pilot prompting indicated that re-issuing the standardized, self-contained prompt within a specialty session produced distinct items without apparent reuse of earlier wording.

In order to have a backup in case of any issues, we obtained two candidate questions from the LLM per topic. If the first version of a question produced by the AI was unsatisfactory (e.g. contained an error or was overly easy or confusing), we substituted it with the second version. The research team—comprising medical content experts and medical educators (*n* = 5)—reviewed all AI-generated outputs for factual accuracy, relevance, and clarity, which reflects a diverse and experienced group of evaluators.

Items were flagged as unsuitable if they contained factual inaccuracies or outdated information, ambiguous wording or more than one potentially correct option, implausible distractors or obvious cues, or clearly inappropriate difficulty (e.g., trivial recall or highly sub-specialized minutiae). The prompt operator (PL) additionally applied a brief standardized quality-control checklist covering adherence to the learning objective, single-best-answer compliance with plausible distractors, absence of ambiguity or leading cues, expected difficulty level, format and style conformity (e.g., avoiding ‘all/none of the above’ or unnecessary negative stems), and potential bias in wording. Items were not substantively edited after LLM generation; aside from removal of a filler word in one item for readability, no changes were made to AI-generated content. After this curation process, the final set of LLM-generated items was approved for use.

Please see Table 2 for an example human question, prompt template, and LLM question. The complete item set (48 MCQs) is provided in the Supplementary Information (Supplementary Data 1).

**Table 2:** Human-authored vs LLM-generated exam question on the same topic, with the prompt used to generate the LLM item (Faculty of Medicine and University Hospital Cologne, University of Cologne; summer 2025)

Factor	Example^a^
Human-authored question	Which of the following best describes oncologic follow-up after radiation therapy? (A) Regular follow-up visits for early detection of tumor recurrence and management of treatment-related adverse effects, as well as contributions to treatment quality assurance. (B) Imaging studies to document tumor size. (C) Evaluation of a reduced-dose radiotherapy regimen to prevent future recurrence. (D) A one-time end-of-therapy examination performed for the statistical reporting of treatment results. (E) Follow-up after radiotherapy includes documenting acute side effects occurring during the course of radiation treatment. Correct answer: A.
Prompt used to generate LLM question^b,c^	Create two multiple-choice questions, each with five answer options and one unambiguously correct answer. Each question should be answerable by both medical students and specialist physicians. The topic of the question is [learning objective]. The question should address [specification], although these terms do not need to appear in every answer option. Ensure that the answer options are not mutually exclusive – that is, different options must not contain directly contradictory statements. Provide the correct answer at the end of each question. In addition, avoid absolute wording and hidden cues, such as “none,” “always,” “exclusively,” or “all.” Ideally, keep the answer options approximately similar in length to avoid unintended cues.
Learning objective specified in prompt	Understanding the tasks and objectives of oncologic follow-up after radiotherapy.
Specification indicated in prompt	Follow-up care: content, objectives, and relevance to patient care and quality assurance.
LLM-generated question	What are the key objectives of follow-up care after radiation therapy? (A) Verifying that radiation therapy was performed in accordance with technical standards (B) Early detection of tumor recurrence and management of treatment-related adverse effects (C) Collecting epidemiological data on the long-term effects of radiation exposure in the population (D) Documenting the delivered radiation dose to meet statutory reporting requirements (E) Coordinating billing procedures within oncology follow-up programs Correct answer: B.

*LLM* large language model, *MCQ* multiple-choice question.

^a^The human-authored items, LLM-generated items, and associated prompts were originally created in German. For Table 2, the examples were translated into English using DeepL (version 25.7.12287188; DeepL SE, Cologne, Germany). A native English-speaking expert subsequently reviewed the translations for accuracy and fluency without altering item content.

^b^Prompts were entered into ChatGPT (GPT-4o-Plus; OpenAI, San Francisco, CA, model release April 25, 2025).

^c^The prompt instructs the model to produce two questions; for display purposes, one representative LLM-generated question is shown here.

### Validation procedures

The final set of 48 questions (24 human-authored and 24 AI-generated) were assembled into a single paper-based questionnaire, which functioned as a mock examination. Items were arranged in a random order, intermixing human- and LLM-origin questions. Participants were blinded to item source; they were not informed of how many or which questions had been generated by the AI.

At the start of the session, participants were informed only that the examination contained experimentally generated questions and that they could optionally attempt to identify each question’s origin. For this purpose, each item on the paper had an adjacent checkbox labeled “human” and “AI”, allowing participants to indicate their guess. Importantly, making this guess was voluntary and had no effect on scoring. No feedback about correctness of the guess was given. All participants, both students and physicians, received identical question booklets. The allotted time for completion was 72 min, corresponding to standard exam timing (approximately 90 s per question). The examination took place in a lecture hall setting for students (and co-attending physicians), supervised by members of the research team to ensure a quiet and academic test environment on 3rd of June, 2025. Physician participants who completed the test separately did so under equivalent conditions (quiet room, same time limit, and no interruptions). For physicians, completion time was captured only opportunistically in a subset and therefore not analyzed.

Throughout the study, participant responses were collected in a pseudonymized manner. Each participant was assigned a unique identification code, which was recorded on the answer sheet instead of their name. The code–name linkage was stored separately from the response data and was not accessible during analysis. This code was later used for participants to privately access their performance results. The completed answer sheets were scanned and scored using the EvaSys evaluation software (EvaSys GmbH). Any scan that produced unclear results was flagged and then manually cross-checked by the study team against the paper form to correct errors.

Students and physicians could use their code to retrieve a summary of their performance (e.g. number of correct answers) and view the correct answers for all questions. This feedback was made available via a secure website displaying pseudonymized results by code, accessible through a QR code provided at the end of the session. All students received the same set of 48 questions and explanatory answers as a study aid after the mock examination, ensuring that no participant was disadvantaged by the research component.

In addition to the participant-based analyses, an expert panel evaluation of item quality was conducted. Four experts, blinded to item origin, independently reviewed 24 of the 48 items per rater after the examination. Items were randomly assigned using an electronic randomization tool and balanced by specialty and origin (eight per specialty—radiation oncology, radiology, nuclear medicine—with four human and four LLM items within each specialty).

The panel consisted of two clinical faculty members (radiation oncology and general medicine) and two medical education specialists (one clinician in internal medicine and one psychologist). This composition provided both specialty-specific and broader pedagogical perspectives, though not full representation of all imaging subdisciplines (e.g., radiology and nuclear medicine). Expert judgments should therefore be interpreted as reflecting general clinical and educational perspectives rather than discipline-specific consensus. Each expert was asked to rate each question on several criteria, including its appropriateness (relevance to the curriculum), didactic quality, and origin judgment. Ratings were given on a Likert-type scale from 1 (low) to 7 (high). A free-text field was available for optional qualitative comments to justify ratings or flag issues.

### Statistical analysis

Item difficulty reflects the proportion of individuals who answered an item correctly. This metric can range from 0 to 1, with higher values—counterintuitively— indicating easier items.

Discriminatory power measures how well an item distinguishes between high- and low-performing students. It is typically reported as the correlation between performance on a given item and the average performance across all other items in a test, and can theoretically range from –1 to +1. To compare the item parameters (i.e., difficulty and discriminatory power) of human-authored versus LLM-generated multiple-choice questions, independent samples t-tests were conducted. Assumptions of normality and homogeneity of variances were assessed using Shapiro–Wilk tests and Levene’s tests, respectively. In cases where the assumption of normality was violated, Mann–Whitney U tests were used as nonparametric alternatives. These analyses were performed for the total sample as well as separately for the subgroups of medical students and physicians.

To assess whether participants could identify the true origin of the questions above chance level, one-sample *t* tests against a fixed reference value of 0.50 were conducted. As in previous analyses, assumptions were checked, and appropriate nonparametric equivalents were applied where necessary. To compare identification performance between medical students and physicians, independent samples t-tests were again employed.

Interrater reliability across the four expert ratings of item content was calculated using the intraclass correlation coefficient (ICC), specifically employing the “single fixed raters” model^32,33^. Additionally, Spearman’s rank-order correlations were used to examine the agreement between the two pairs of raters (i.e., clinical experts and medical education experts). To assess interrater agreement regarding the identification of item origin, Fleiss’ Kappa was calculated. Statistical analyses and data visualizations were conducted using R (R Core Team, 2022), RStudio (Posit Team, 2024), and Microsoft Excel (Microsoft Corp, 2021).

## Supplementary information

Supplementary information

## Data Availability

All datasets generated or analyzed during this study are not publicly available due to institutional and participant privacy considerations but may be made available to qualified researchers upon reasonable request to the corresponding author. The preregistered protocol is available at the Open Science Framework (OSF; https://osf.io/d67fy). All item stems, prompts, and answer keys are provided in the Supplementary Information.

## Code availability

The underlying code used in this study is not publicly available but may be provided to qualified researchers upon reasonable request to the corresponding author.

## References

[1] Lu, Z. et al. Large language models in biomedicine and health: current research landscape and future directions. *J. Am. Med Inf. Assoc.* **31**, 1801–1811 (2024). 10.1093/jamia/ocae202 PMC11339542 39169867

[2] Singhal, K. et al. Large language models encode clinical knowledge. *Nature* **620**, 172–180 (2023). 10.1038/s41586-023-06291-2 PMC10396962 37438534

[3] Huang, Y. et al. Principles of artificial intelligence in radiooncology. *Strahlenther. Onkol.* **201**, 210–235 (2025). 10.1007/s00066-024-02272-0 PMC11839771 39105746

[4] Decuyper, M., Maebe, J., Van Holen, R. & Vandenberghe, S. Artificial intelligence with deep learning in nuclear medicine and radiology. *EJNMMI Phys.* **8**, 81 (2021). 10.1186/s40658-021-00426-y PMC8665861 34897550

[5] Busch, F. et al. Large language models for structured reporting in radiology: Past, present, and future. *Eur. Radio.* **35**, 2589–2602 (2025). 10.1007/s00330-024-11107-6 PMC12021971 39438330

[6] Lammert, J. et al. Expert-guided large language models for clinical decision support in precision oncology. *JCO Precis. Oncol.* **8**, e2400478 (2024). 10.1200/PO-24-00478 39475661

[7] Ong, J. C. L. et al. Ethical and regulatory challenges of large language models in medicine. *Lancet Digit Health* **6**, e428–e432 (2024). 10.1016/S2589-7500(24)00061-X 38658283

[8] Boscardin, C. K., Gin, B., Golde, P. B. & Hauer, K. E. ChatGPT and Generative Artificial Intelligence for Medical Education: Potential Impact and Opportunity. *Acad. Med.* **99**, 22–27 (2024). 10.1097/ACM.0000000000005439 37651677

[9] Gilardi, F., Alizadeh, M. & Kubli, M. ChatGPT outperforms crowd workers for text-annotation tasks. *Proc. Natl. Acad. Sci. USA* **120**. 10.1073/pnas.2305016120 (2023). PMC10372638 37463210

[10] Homolak, J. Opportunities and risks of ChatGPT in medicine, science, and academic publishing: a modern Promethean dilemma. *Croat. Med. J.* **64**, 1–3 (2023). 10.3325/cmj.2023.64.1 PMC10028563 36864812

[11] Karpicke, J. D. & Roediger, H. L. 3rd The critical importance of retrieval for learning. *Science* **319**, 966–968 (2008). 10.1126/science.1152408 18276894

[12] Pan, S. C. & Rickard, T. C. Transfer of test-enhanced learning: a meta-analytic review and synthesis. *Psychol. Bull.* **144**, 710–756 (2018). 10.1037/bul0000151 29733621

[13] Luo, R. et al. BioGPT: generative pre-trained transformer for biomedical text generation and mining. *Brief. Bioinform* **23**, bbac409 (2022). 10.1093/bib/bbac409 36156661

[14] Gao, C. A. et al. Comparing scientific abstracts generated by ChatGPT to real abstracts with detectors and blinded human reviewers. *npj Digit Med.* **6**, 75 (2023). 10.1038/s41746-023-00819-6 PMC10133283 37100871

[15] Stadler, M., Horrer, A. & Fischer, M. R. Crafting medical MCQs with generative AI: a how-to guide on leveraging ChatGPT. *GMS J. Med. Educ.* **41**, Doc20 (2024). 10.3205/zma001675 PMC11106576 38779693

[16] Ramadan, S. et al. Evaluating ChatGPT’s competency in radiation oncology: A comprehensive assessment across clinical scenarios. *Radiother. Oncol.* **202**, 110645 (2025). 10.1016/j.radonc.2024.110645 39571686

[17] Moor, M. et al. Foundation models for generalist medical artificial intelligence. *Nature* **616**, 259–265 (2023). 10.1038/s41586-023-05881-4 37045921

[18] Laupichler, M. C., Aster, A., Schirch, J. & Raupach, T. Artificial intelligence literacy in higher and adult education: A scoping literature review. Comput. *Educ. Artif. Intell.* **3**, 100101 (2022).

[19] Tolsgaard, M. G. et al. The fundamentals of artificial intelligence in medical education research: AMEE Guide No. 156. *Med Teach.* **45**, 565–573 (2023). 10.1080/0142159X.2023.2180340 36862064

[20] Laupichler, M. C., Rother, J. F., Grunwald Kadow, I. C., Ahmadi, S. & Raupach, T. Large language models in medical education: comparing ChatGPT- to human-generated exam questions. *Acad. Med* **99**, 508–512 (2024). 10.1097/ACM.0000000000005626 38166323

[21] Mistry, N. P. et al. Large language models as tools to generate radiology board-style multiple-choice questions. *Acad. Radiol.* **31**, 3872–3878 (2024). 10.1016/j.acra.2024.06.046 39013736

[22] Camarata, T. et al. LLM-generated multiple-choice practice quizzes for preclinical medical students. *Adv. Physiol. Educ.* **49**, 758–763 (2025). 10.1152/advan.00106.2024 40516963

[23] Grévisse, C., Pavlou, M. A. S. & Schneider, J. G. Docimological quality analysis of LLM-generated multiple-choice questions in computer science and medicine. *SN Comput. Sci.* **5**, 636 (2024).

[24] Kıyak, Y. S., Górski, S., Tokarek, T., Pers, M., & Kononowicz, A. A. Large language models for generating key-feature questions in medical education. *Med. Educ. Online*, **30**. 10.1080/10872981.2025.2574647 (2025). PMC12573569 41159362

[25] Yapıcı Coşkun, Z., Kıyak, Y. S., Coşkun, Ö, Budakoğlu, I. İ & Özdemir, Ö Large language models for generating script concordance test in obstetrics and gynecology: ChatGPT and Claude. *Med. Teach.* **47**, 1767–1771 (2025). 10.1080/0142159X.2025.2497888 40305090

[26] Artsi, Y. et al. Large language models for generating medical examinations: systematic review. *BMC Med Educ.* **24**, 354 (2024). 10.1186/s12909-024-05239-y PMC10981304 38553693

[27] Özer, N. E., Balcı, Y., Bölükbaşı, G., İlhan, B., & Güneri, P. Examining the Role of Artificial Intelligence in Assessment: A Comparative Study of ChatGPT and Educator-Generated Multiple-Choice Questions in a Dental Exam. *Eur. J. Dental Educ.* 10.1111/eje.70034 (2025). PMC13383294 40785272

[28] Chauhan, A., Khaliq, F. & Nayak, K. R. Assessing quality of scenario-based multiple-choice questions in physiology: Faculty-generated vs. chatgpt-generated questions among phase I medical students. *Int. J. Artif. Intell. Educ*. 10.1007/s40593-025-00471-z (2025).

[29] Kondo, T., Okamoto, M. & Kondo, Y. Pilot study on using large language models for educational resource development in Japanese radiological technologist exams. *Med. Sci. Educ.* **35**, 919–927 (2025). 10.1007/s40670-024-02251-1 PMC12059199 40353040

[30] Shrout, P. E. & Fleiss, J. L. Intraclass correlations: Uses in assessing rater reliability. *Psychol. bull.* **86**, 420 (1979). 10.1037//0033-2909.86.2.420 18839484

[31] Joncas, S. X., St-Onge, C., Bourque, S. & Perspect, P. Re-using questions in classroom-based assessment: an exploratory study at the undergraduate medical education level. *Perspect. Med. Educ.* **7**, 373–378 (2018). 10.1007/s40037-018-0482-1 PMC6283783 30421331

[32] Appelhaus, S., Werner, S., Grosse, P. & Kämmer, J. E. Feedback, fairness, and validity: effects of disclosing and reusing multiple-choice questions in medical schools. *Med. Educ. Online* **28**, 2143298 (2022). 10.1080/10872981.2022.2143298 PMC9662023 36350605

[33] Ragaller, S. V., Rother, J. F., Aster, A. & Raupach, T. Study habits in medical education: examining how German medical students study using a cross-sectional mixed-methods survey. *Med. Sci. Educ.* **35**, 1441–1449 (2025). 10.1007/s40670-025-02324-9 PMC12228932 40625967

[34] Maren, M., Himmelbauer, M., Boldt, K. & Oksche, A. Legal aspects of generative artificial intelligence and large language models in examinations and theses. *GMS J. Med. Educ.* **41**, Doc47 (2024). 10.3205/zma001702 PMC11474642 39415812

[35] Karabacak, M. & Margetis, K. Embracing large language models for medical applications: opportunities and challenges. *Cureus* **15**, e39305 (2023). 10.7759/cureus.39305 PMC10292051 37378099

[36] Downing, S. M. Reliability: on the reproducibility of assessment data. *Med Educ.* **38**, 1006–1012 (2004). 10.1111/j.1365-2929.2004.01932.x 15327684

[37] Jozefowicz, R. F. et al. The quality of in-house medical school examinations. *Acad. Med* **77**, 156–161 (2002). 10.1097/00001888-200202000-00016 11841981

[38] Tarrant, M., Knierim, A., Hayes, S. K. & Ware, J. The frequency of item-writing flaws in multiple-choice questions used in high-stakes nursing assessments. *Nurse Educ. Today* **26**, 662–671 (2006). 10.1016/j.nedt.2006.07.006 17014932

[39] Faherty, A., Counihan, T., Kropmans, T. & Finn, Y. Inter-rater reliability in clinical assessments: do examiner pairings influence candidate ratings?. *BMC Med Educ.* **20**, 147 (2020). 10.1186/s12909-020-02009-4 PMC7212618 32393228

[40] Govaerts, M. J., Schuwirth, L. W., van der Vleuten, C. P. & Muijtjens, A. M. Workplace-based assessment: effects of rater expertise. *Adv. Health Sci. Educ.* **16**, 151–165 (2011). 10.1007/s10459-010-9250-7 PMC3068251 20882335

[41] Downing, S. M. The effects of violating standard item-writing principles on tests and students: the consequences of using flawed test items on achievement examinations in medical education. *Adv. Health Sci. Educ.* **10**, 133–143 (2005). 10.1007/s10459-004-4019-5 16078098

[42] Ning, Y. et al. Generative artificial intelligence and ethical considerations in health care: a scoping review and ethics checklist. *Lancet Digit Health* **6**, e848–e856 (2024). 10.1016/S2589-7500(24)00143-2 PMC11542614 39294061

[43] Hauer, K. E., Park, Y. S., Bullock, J. L. & Tekian, A. “My assessments are biased!” Measurement and sociocultural approaches to achieve fairness in assessment in medical education. *Acad. Med.* **98**, S16–S27 (2023). 10.1097/ACM.0000000000005245 37094278

[44] Hope, D., Adamson, K., McManus, I. C., Chis, L. & Elder, A. Using differential item functioning to evaluate potential bias in a high-stakes postgraduate knowledge-based assessment. *BMC Med. Educ.* **18**, 64 (2018). 10.1186/s12909-018-1143-0 PMC5883583 29615016

[45] Caliskan, A., Bryson, J. J. & Narayanan, A. Semantics derived automatically from language corpora contain human-like biases. *Science* **356**, 183–186 (2017). 10.1126/science.aal4230 28408601

[46] Gichoya, J. W. et al. AI recognition of patient race in medical imaging: a modelling study. *Lancet Digit Health* **4**, e406–e414 (2022). 10.1016/S2589-7500(22)00063-2 PMC9650160 35568690

[47] Meskó, B. & Topol, E. J. The imperative for regulatory oversight of large language models (or generative AI) in healthcare. *npj Digit Med.* **6**, 120 (2023). 10.1038/s41746-023-00873-0 PMC10326069 37414860

[48] World Medical Association World Medical Association Declaration of Helsinki: Ethical principles for medical research involving human subjects. *JAMA* **310**, 2191–2194 (2013). 10.1001/jama.2013.281053 24141714

[49] von Elm, E. et al. The strengthening the reporting of observational studies in epidemiology (STROBE) statement: Guidelines for reporting observational studies. *Lancet* **370**, 1453–1457 (2007). 10.1016/S0140-6736(07)61602-X 18064739

[50] Medizinischer Fakultätentag der Bundesrepublik Deutschland (MFT). Nationaler Kompetenzbasierter Lernzielkatalog Medizin (NKLM), Version 2.0. 2015. https://nklm.de/menu (Accessed 25 October 2025).

