# Supplementary material for: Psychometric properties and detectability of GPT-4o–generated multiple-choice questions compared with human-authored items across imaging specialties
Source: NPJ Digit Med. 2026 Jan 8;9:132. doi: 10.1038/s41746-025-02313-7 (PMC12881591; doi:10.1038/s41746-025-02313-7)
Supplement: Supplementary file 1 — Supplementary information [file 41746_2025_2313_MOESM1_ESM.pdf]

Supplementary Information (S)

S 1 → Question lists

The human-authored items, LLM-generated items, and associated prompts were originally created in German. For S1, the examples were translated into English using DeepL (version 25.7.12287188; DeepL SE, Cologne, Germany). A native English-speaking expert subsequently reviewed the translations for accuracy and fluency without altering item content.

Question list – Layout 1 (Human-authored)

	**Layout 1**<br><br>***(correct answer underlined)***	**Associated teaching session incl. materials *(if available)***	**[learning objective] + [specification]**	**Notes *(optional)***	**Order after randomization (ID)**
1<br><br>RA	Which imaging modality should be preferred in pediatric patients, considering radiation safety and cost, when clinically appropriate?<br><br>(A) Magnetic resonance<br><br>imaging (MRI)<br><br>(B) Conventional<br><br>radiography (x-ray)<br><br><u>(C) Ultrasound</u><br><br>(D) Scintigraphy<br><br>(E) Computed<br><br>tomography (CT)	lecture; seminar	Learning objective: overview of potential imaging modalities in pediatric radiology and their cost–benefit profile.<br><br>Specification: prioritization of modalities based on radiation protection (dose considerations) and economic factors.		22

2<br>RA	For which patient does MRI require especially careful risk–benefit assessment?<br><br>(A) Patient with a pacemaker<br><br>(B) Patient with a known allergy to iodinated contrast media<br><br>(C) Patient with a titanium bone screw<br><br>(D) Patient with chronic hypertension<br><br>(E) Patient with prior x-ray examinations	lecture	Learning objective: patient safety in diagnostic imaging.<br><br>Specification: identifying contraindications to magnetic resonance imaging (MRI).		20
3<br>RA	Which of the following is **not** an imaging sign of spondylodiscitis?<br><br>(A) Low signal of the intervertebral disc on fluid-sensitive MRI sequences in the setting of disc liquefaction<br><br>(B) Reactive osteitis of the adjacent vertebral bodies on MRI with intraosseous contrast enhancement<br><br>(C) Paravertebral soft-tissue “rind”/phlegmon<br><br>(D) Erosions and irregularity of the adjacent endplates	seminar	Learning objective: musculoskeletal imaging in inflammatory conditions.<br><br>Specification: recognizing the radiologic signs of spondylodiscitis and how they present across imaging modalities.		18

	(E) Abscess formation extending intraspinally or into the psoas muscle				
4<br>RA	Which of the following is generally **not** a complication requiring treatment after osteosynthesis?<br><br>(A) Implant loosening<br><br>(B) Periprosthetic fracture<br><br>(C) Nonunion (pseudarthrosis)<br><br>(D) Physeal (growth-plate) injury<br><br>(E) Callus formation	seminar	Learning objective: trauma imaging (radiology).<br><br>Specification: recognizing potential complications detectable on radiologic imaging after trauma.		26

5<br><br>RA	Which procedure is **not** part of interventional oncology?<br><br>(A) Transarterial chemoembolization (TACE)<br><br>(B) Percutaneous microwave ablation (MWA)<br><br>(C) Irreversible electroporation (IRE)<br><br>(D) <u>Radiosynoviorthesis</u><br><br>(E) Percutaneous cryoablation	seminar	Learning objective: interventional oncology in radiology.<br><br>Specification: identifying appropriate interventional radiology procedures for tumor treatment.		37
6<br><br>RA	In which imaging examination can barium-based contrast be used?<br><br>(A) <u>Fluoroscopy (GI contrast study)</u><br><br>(B) MR enterography (Sellink)<br><br>(C) Contrast-enhanced ultrasound (CEUS)<br><br>(D) Computed tomography (CT)<br><br>(E) Left lateral decubitus abdominal radiograph	seminar	Learning objective: use of contrast media in radiology.<br><br>Specification: selecting and administering indication-specific and modality-specific contrast media.		9

7<br><br>RA	Which statement is **not** correct in a systematic interpretation of a chest radiograph?<br><br>(A) Any indwelling devices should be checked for possible malposition.<br><br>(B) <u>Careful inspection of the costophrenic angles helps to rule out hilar lymphadenopathy.</u><br><br>(C) Pleural dehiscence (visceral–parietal pleural separation) is often associated with pneumothorax.<br><br>(D) Widening of the mediastinal silhouette can be tumor-related.<br><br>(E) A relatively higher right hemidiaphragm is very common.	lecture; seminar	Learning objective: systematic interpretation of a chest radiograph (CXR).<br><br>Specification: recognizing common chest radiograph (CXR) pathologies and their radiographic appearances.		46

8<br><br>RA	Which of the following neuroradiological interventions is **not** catheter-based?<br><br>(A) Mechanical thrombectomy for acute ischemic stroke<br><br>(B) <u>Periradicular pain therapy (PRT)</u><br><br>(C) Endovascular aneurysm treatment (coiling/flow diversion)<br><br>(D) Embolization of a vascular malformation<br><br>(E) Carotid dissection stenting	lecture; seminar	Learning objective: knowledge of neuroradiological interventional options.<br><br>Specification: fundamental techniques of neurointerventional procedures.		36
1<br><br>NM	A 66-year-old woman presents with laboratory-confirmed subclinical hyperthyroidism. Which finding on 99mTc-pertechnetate thyroid scintigraphy most strongly suggests unifocal thyroid autonomy?<br><br>(A) Uniform, diffuse increased uptake in both lobes<br><br>(B) Focally absent uptake in the left lobe<br><br>(C) Multiple hypofunctioning areas in both lobes		Learning objective: interpretation of thyroid scintigraphy with Tc-99m (pertechnetate); understanding how radionuclide uptake patterns inform the diagnosis of thyroid dysfunction.<br><br>Specification: identifying the association between focal increased uptake (“hot spot”) on scintigraphy and (uni)focal thyroid autonomy.		44

	(D) Focal increased uptake in the right lobe<br><br>(E) A unifocal autonomy cannot be detected on 99mTc scintigraphy				
2<br><br>NM	The EMA (European Medicine Agency) approved lutetium-177 PSMA-617 under the trade name Pluvicto® for the treatment of prostate cancer in 2022.<br><br>In which clinical scenario is this therapy currently indicated?<br><br>(A) in the metastatic hormone-sensitive stage.<br><br>(B) in the metastatic castration-resistant stage after treatment with a modern androgen receptor pathway inhibitor (e.g., enzalutamide) and BRCA wild type.<br><br>(C) in the metastatic castration-resistant stage with BRCA wild type after treatment with a modern androgen receptor pathway inhibitor and after first-line chemotherapy (docetaxel).<br><br>(D) in the initial stage after radical prostatectomy in the presence of a high-risk prostate carcinoma.		Learning objective: knowledge of the clinical use of novel radiopharmaceuticals for prostate cancer therapy, exemplified by Lu-177–PSMA-617 (Pluvicto; approved 2022).<br><br>Specification: identifying the precise indication/eligibility criteria for Lu-177–PSMA radioligand therapy.		23

	(E) in biochemical recurrence after initial curative-intent therapy.				
3<br><br>NM	Which assignment of a disease to a nuclear medicine therapy is **incorrect**?<br><br>(A) Thyroid carcinoma – radioiodine therapy (I-131)<br><br>(B) Neuroendocrine tumor – Lu-177 peptide receptor radionuclide therapy<br><br>(C) Prostate carcinoma – Lu-177 PSMA-ligand therapy<br><br>(D) <u>Neuroblastoma – phosphorus-32 particles via probe technique</u><br><br>(E) Hepatocellular carcinoma – selective internal radiotherapy with Y-90 particles		Learning objective: knowledge of common radionuclide therapies.<br><br>Specification: recognizing mismatches between a radionuclide therapy and its appropriate radiopharmaceutical.		34
4<br><br>NM	Which of the following **cannot** be established with sufficient reliability using a nuclear medicine technique?<br><br>(A) Demonstration of a renal parenchymal (cortical) defect on 99mTc-DMSA renal scintigraphy		Learning objective: recall and articulate the capabilities and limitations of commonly used nuclear medicine techniques.<br><br>Specification: determine when a stated diagnostic finding cannot be obtained with the specified nuclear medicine modality (i.e., recognize		5

	(B) Demonstration of urinary outflow obstruction with 99mTc-MAG3 renography<br><br>(C) Demonstration of exercise-induced myocardial ischemia with myocardial perfusion scintigraphy<br><br>(D) Demonstration of thyroid carcinoma with 99mTc-MIBI scintigraphy<br><br>(E) Demonstration of melanoma spread to regional sentinel lymph nodes using lymphoscintigraphy followed by excision and histopathology		non-feasible detection with that technique).		
5<br><br>NM	Which classification system is used for the ultrasound assessment of malignancy risk in thyroid nodules?<br><br>(A) BI-RAS<br><br>(B) TNM<br><br>(C) TI-RAS<br><br>(D) BOSNIAK<br><br>(E) Perugini score		Learning objective: knowledge of classification systems for thyroid tumors.<br><br>Specification: recall and state the appropriate classification system to use in the context of thyroid ultrasound.		47

6<br><br>NM	Which radiotracer is routinely available in Germany for brain PET (“brain PET”) in dementia diagnostics?<br><br>(A) 18F-fluorodeoxyglucose (FDG)<br><br>(B) 18F-fluoroethyl-tyrosine (FET)<br><br>(C) 68Ga-fibroblast activation protein inhibitor (FAPI)<br><br>(D) 18F-lecanemab<br><br>(E) 68Ga-donanemab		Learning objective: knowledge of PET tracers used in nuclear medicine diagnostics (positron-emitting radiopharmaceuticals).<br><br>Specification: recall and state an appropriate tracer for brain PET in the diagnostic workup of dementia.		39
7<br><br>NM	Which statement about Parkinson’s diagnostics using single-photon emission computed tomography (SPECT) is correct?<br><br>(A) I-123-FP-CIT (DaTSCAN) is suitable for assessing the integrity of presynaptic dopaminergic terminals in the striatum.<br><br>(B) I-123-FP-CIT (DaTSCAN) mainly binds to postsynaptic dopamine transporters in the substantia nigra.<br><br>(C) In Parkinson’s disease there is primarily degeneration of cortical serotonergic neurons, whose density		Learning objective: knowledge of tracers used for diagnosing Parkinson’s disease with SPECT (single-photon emission computed tomography).<br><br>Specification: recall and state the correct tracer and its mechanism of action in Parkinson diagnostics.		48

	can be assessed with I-123-FP-CIT SPECT.<br><br>(D) In I-123-FP-CIT SPECT one exploits beta-plus decay of I-123 with coincidence detection using ring detectors.<br><br>(E) I-123-FP-CIT (DaTSCAN) is purely experimental and not available in routine care in Germany.				
8<br><br>NM	Which combination correctly matches the PET tracer with its uptake principle/diagnostic target?<br><br>(A) 68Ga-PSMA – prostate-specific membrane antigen<br><br>(B) 68Ga-DOTATATE – somatostatin receptor<br><br>(C) 18F-FDG (fluorodeoxyglucose) – glucose metabolism<br><br>(D) 18F-FET (fluoroethyl-tyrosine) – amino acid metabolism<br><br>(E) <u>All answers are correct</u>		Learning objective: knowledge of the uptake characteristics of key positron emission tomography (PET) tracers.<br><br>Specification: recall and state the correct pairings of selected tracers with their underlying uptake mechanisms.		29

1<br><br>RO	In the context of radiotherapy, what does tumor follow-up most closely entail?<br><br>(A) Regular surveillance to detect recurrences early, monitor treatment-related toxicities, and ensure quality of care.<br><br>(B) Imaging studies to document tumor size.<br><br>(C) Evaluation of a reduced-dose radiotherapy regimen to prevent future recurrences.<br><br>(D) A one-time post-treatment visit to collect outcome statistics.<br><br>(E) Documentation of acute side effects during irradiation.	practical course; lecture	Learning objective: understanding the tasks and objectives of oncologic follow-up after radiotherapy.<br><br>Specification: follow-up care: content, objectives, and relevance to patient care and quality assurance.		17

2<br>RO	What are the three pillars of cancer treatment?<br><br>(A) Pain management, nutritional counseling, and psychotherapy<br><br>(B) Immunotherapy, physiotherapy, and rehabilitation<br><br>(C) <u>Surgery, radiotherapy, and systemic therapy</u><br><br>(D) Genetic counseling, palliative care, and radiology<br><br>(E) Antibody therapy, hormone therapy, and blood transfusion	practical course	Learning objective: fundamentals of oncologic treatment concepts.<br><br>Specification: the three classical pillars of cancer therapy – surgery, radiotherapy, and systemic therapy.		4

3<br><br>RO	What is a typical therapeutic advantage of the concurrent use of chemotherapy and radiotherapy in malignant tumors?<br><br>(A) Chemotherapy neutralizes the DNA damage caused by radiation.<br><br>(B) Radiation prevents the systemic distribution of cytotoxic drugs.<br><br>(C) <u>Combined therapy can make tumor cells more sensitive to radiation.</u><br><br>(D) The radiation dose can be doubled without increasing the risk of side effects.<br><br>(E) Chemotherapy shortens the required treatment time through complete tumor necrosis.	practical course	Learning objective: fundamentals of radiation oncology – chemoradiation and combination strategies.<br><br>Specification: understanding the radiosensitizing effect of concurrent chemoradiotherapy.		42
4<br><br>RO	Which dose is expressed in gray (Gy)?<br><br>(A) Equivalent dose<br><br>(B) Effective dose<br><br>(C) Biological half-life<br><br>(D) Exposure dose<br><br>(E) <u>Energy dose</u>	practical course; lecture	Learning objective: radiation protection – physical foundations of dosimetry.<br><br>Specification: knowledge of dose quantities and units used in radiology.		15

5<br>RO	Which phases of the cell cycle are the most radiosensitive?<br><br>(A) Mitotic and G2 phase<br><br>(B) G1 and early S phase<br><br>(C) Late S and G1 phase<br><br>(D) G0 and G1 phase<br><br>(E) S phase and interphase	lecture	Learning objective: radiation biology – cell-cycle dependence of radiosensitivity.<br><br>Specification: radiosensitivity varies across cell-cycle phases with respect to ionizing-radiation–induced DNA damage.		30
6<br>RO	How do overall treatment time and interruptions in radiotherapy affect tumor control?<br><br>(A) A prolonged overall treatment time generally improves tumor control due to better tolerability.<br><br>(B) Treatment interruptions are uncritical as long as the total dose is delivered.<br><br>(C) Short breaks in the course can increase tumor control because they allow more normal-tissue recovery.	practical course	Learning objective: impact of radiotherapy parameters on tumor control.<br><br>Specification: the role of total treatment time and unplanned interruptions in curative radiotherapy.		3

	(D) For the same total dose, a long overall treatment time and treatment breaks worsen tumor control.<br><br>(E) The radiobiological effect occurs at the end of the course and is independent of time.				
7<br><br>RO	In radiotherapy, thermoplastic masks are used for certain disease sites. What is their purpose?<br><br>(A) They immobilize the target region and enable a reproducible treatment setup.<br><br>(B) They reduce the skin dose through reflective surfaces.<br><br>(C) They improve temperature regulation of the irradiated area during the session.<br><br>(D) They protect adjacent organs by targeted shielding.<br><br>(E) They replace image-guided positioning verification during planning.	practical course	Learning objective: fundamentals of radiotherapy – patient positioning and treatment planning.<br><br>Specification: function and purpose of thermoplastic masks for immobilization and treatment field setup.		40

8<br><br>RO	You are treating a 70-year-old man with prostate cancer using external-beam radiotherapy. Which chronic adverse effect is most likely?<br><br>(A) Scalp alopecia<br><br>(B) Urinary incontinence<br><br>(C) Radiation dermatitis of the scrotum<br><br>(D) Marked nausea and vomiting<br><br>(E) Hand–foot syndrome	practical course; lecture	Learning objective: recognize and contextualize long-term effects of radiotherapy in malignant disease.<br><br>Specification: radiotherapy for genitourinary tumors – adverse and late effects.		**28**

Question list – Layout 1 (LLM-generated)

	Layout 2<br><br>*(correct answer underlined)*	Associated teaching session incl. materials *(if available)*	[learning objective] + [specification]	Notes *(optional)*	Order after randomization (ID)
1<br><br>RA	When selecting imaging in pediatric radiology, both radiation exposure and cost matter. Which statement best reflects an appropriate prioritization for nonspecific abdominal pain in children?<br><br>(A) CT should be preferred because it is fast and provides high image quality.<br><br>(B) <u>Ultrasound is a sensible first option because it involves no ionizing radiation and is cost-effective.</u><br><br>(C) MRI should be used initially because it requires no contrast and suits all questions.<br><br>(D) An abdominal x-ray is the best choice to reliably exclude inflammation.<br><br>(E) The choice should primarily be based on insurer reimbursement.	lecture; seminar	Learning objective: overview of potential imaging modalities in pediatric radiology and their cost–benefit profile.<br><br>Specification: prioritization of modalities based on radiation protection (dose considerations) and economic factors.		43

2<br><br>RA	Which of the following details from the patient interview should be checked particularly carefully before MRI because it could represent a relevant contraindication?<br><br>(A) Permanent dental fillings containing metal<br><br>(B) Status post laparoscopic cholecystectomy more than one year ago<br><br>(C) Implantation of a pacemaker with unknown MR compatibility<br><br>(D) Oral iron supplementation for one week<br><br>(E) Status post total knee arthroplasty with complete wound healing	lecture	Learning objective: patient safety in diagnostic imaging.<br><br>Specification: identifying contraindications to magnetic resonance imaging (MRI).		12

3<br><br>RA	Which radiological finding most strongly supports the early suspected diagnosis of spondylodiscitis on MRI?<br><br>(A) Osteophytic spurs at multiple vertebral bodies<br><br>(B) Decreased T2 signal of the intervertebral disc<br><br>(C) Contrast enhancement of the disc with adjacent bone marrow edema<br><br>(D) Mild loss of vertebral body height with preserved disc structure<br><br>(E) Calcifications in the disc region without soft-tissue reaction	seminar	Learning objective: musculoskeletal imaging in inflammatory conditions.<br><br>Specification: recognizing the radiologic signs of spondylodiscitis and how they present across imaging modalities.		38

4<br><br>RA	A patient presents after an open tibial fracture with persistent pain and swelling. Which radiologic finding is most consistent with post-traumatic osteomyelitis?<br><br>(A) Subtle fracture line with perifracture bone-marrow edema<br><br>(B) Soft-tissue densification without visible bone reaction<br><br>(C) <u>Cortical bone defect with adjacent periosteal reaction and soft-tissue edema</u><br><br>(D) Regular fracture healing with homogeneous callus formation<br><br>(E) Knee joint effusion nearby without osseous changes	seminar	Learning objective: trauma imaging (radiology).<br><br>Specification: recognizing potential complications detectable on radiologic imaging after trauma.		24

5<br><br>RA	In a patient with unresectable hepatocellular carcinoma (HCC) and preserved liver function (Child–Pugh A), a local therapy is being considered. Which interventional radiology procedure is most appropriate under these conditions?<br><br>(A) Systemic radiochemotherapy followed by surgical resection<br><br>(B) Percutaneous drainage to decompress the biliary tree<br><br>(C) <u>Transarterial chemoembolization (TACE) for local tumor control</u><br><br>(D) Endoscopic marking to prepare for radiotherapy<br><br>(E) Selective hepatic venography to assess metastases	seminar	Learning objective: interventional oncology in radiology.<br><br>Specification: identifying appropriate interventional radiology procedures for tumor treatment.		2

6<br>RA	Which contrast-medium setup is typically appropriate for CT angiography of the thoracic aorta to assess a dissection?<br><br>(A) Oral barium contrast to opacify the stomach lumen<br><br>(B) Intravenous gadolinium-based contrast<br><br>(C) Air insufflation via nasal tube to opacify the esophagus<br><br>(D) <u>Intravenous iodinated contrast with bolus-triggered acquisition</u><br><br>(E) Ultrasound contrast agent to display heart rate	seminar	Learning objective: use of contrast media in radiology.<br><br>Specification: selecting and administering indication-specific and modality-specific contrast media.		7

7<br>RA	Which radiological constellation on a chest radiograph most strongly suggests a pneumothorax?<br><br>(A) Homogeneous opacification of one hemithorax with mediastinal shift<br><br>(B) Increased lucency without visible vascular markings to the thoracic periphery<br><br>(C) Bilateral reticulonodular pattern with basal predominance<br><br>(D) Streaky opacities along the bronchi with central predominance<br><br>(E) Diffuse opacification with an indistinct cardiac silhouette	lecture; seminar	Learning objective: systematic interpretation of a chest radiograph (CXR).<br><br>Specification: recognizing common chest radiograph (CXR) pathologies and their radiographic appearances.		31

8<br><br>RA	Which technique is typically used for mechanical thrombectomy in acute stroke therapy?<br><br>(A) Intra-arterial administration of calcium-channel blockers via the carotid artery<br><br>(B) Balloon angioplasty of the middle cerebral artery<br><br>(C) <u>Aspiration or stent retriever to remove the intracranial thrombus</u><br><br>(D) Local cryotherapy in the region of the circle of Willis<br><br>(E) Endoscopic thrombus removal via a transorbital route	lecture; seminar	Learning objective: knowledge of neuroradiological interventional options.<br><br>Specification: fundamental techniques of neurointerventional procedures.		10

1<br><br>NM	In a thyroid scan with 99mTc-pertechnetate, which statement is most accurate when a focal area shows markedly increased tracer uptake?<br><br>(A) The finding most likely indicates an inflammatory process in that area.<br><br>(B) This typically represents a “cold” nodule with increased malignancy risk.<br><br>(C) <u>The focal hyperuptake suggests a focally autonomous thyroid function (“hot” nodule).</u><br><br>(D) This scintigraphic technique is primarily suited to detect thyroid carcinoma.<br><br>(E) The activity accumulation often points to suppressed TSH secretion in hypothyroidism.		Learning objective: interpretation of thyroid scintigraphy with Tc-99m (pertechnetate); understanding how radionuclide uptake patterns inform the diagnosis of thyroid dysfunction.<br><br>Specification: identifying the association between focal increased uptake (“hot spot”) on scintigraphy and (uni)focal thyroid autonomy.		21

2<br><br>NM	Which of the following clinical situations most closely corresponds to the currently approved indication for radionuclide therapy with Lu-177 PSMA?<br><br>A) Patients with hormone-naïve prostate cancer and isolated extracapsular extension.<br><br>(B) Patients with metastatic castration-resistant prostate cancer after prior therapy with an androgen receptor pathway inhibitor and taxane-based chemotherapy.<br><br>(C) Patients with locally advanced prostate cancer who have so far received only androgen-deprivation therapy.<br><br>(D) Patients with biochemical recurrence after radical prostatectomy and negative imaging.<br><br>(E) Patients with bone metastases while on ongoing primary androgen-deprivation therapy without additional prior treatments.		Learning objective: knowledge of the clinical use of novel radiopharmaceuticals for prostate cancer therapy, exemplified by Lu-177–PSMA-617 (Pluvicto; approved 2022).<br><br>Specification: identifying the precise indication/eligibility criteria for Lu-177–PSMA radioligand therapy.		6

3<br><br>NM	Which pairing of radionuclide therapy and agent is **incorrect**?<br><br>(A) Radioiodine therapy for differentiated thyroid carcinoma – iodine-131<br><br>(B) Radionuclide therapy for neuroendocrine tumors – lutetium-177 DOTATATE<br><br>(C) Radiosynoviorthesis for knee osteoarthritis – rhenium-186<br><br>(D) Radionuclide therapy for liver metastases of colorectal cancer – yttrium-90 microspheres<br><br>(E) <u>Radionuclide therapy for osseous prostate cancer – technetium-99m HDP</u>		Learning objective: knowledge of common radionuclide therapies.<br><br>Specification: recognizing mismatches between a radionuclide therapy and its appropriate radiopharmaceutical.		1

4<br><br>NM	Which of the following diagnostic statements is **least** reliably established with conventional skeletal scintigraphy using 99mTc-HDP?<br><br>(A) Assessing the activity of degenerative joint changes<br><br>(B) Detecting osseous metastases in prostate cancer<br><br>(C) <u>Differentiating between inflammatory and neoplastic causes of a bone lesion</u><br><br>(D) Demonstrating increased bone turnover during fracture healing<br><br>(E) Showing focal uptake in the spine when spondylitis is suspected		Learning objective: recall and articulate the capabilities and limitations of commonly used nuclear medicine techniques.<br><br>Specification: determine when a stated diagnostic finding cannot be obtained with the specified nuclear medicine modality (i.e., recognize non-feasible detection with that technique).		35

5<br><br>NM	Which system is used to categorize thyroid nodules based on sonographic criteria with the aim of estimating the risk of malignancy?<br><br>(A) TIRAS system, with assessment based on criteria such as echogenicity, margins, and calcifications<br><br>(B) TNM system, which primarily considers the size of the nodule and the presence of distant metastases<br><br>(C) Bethesda classification, focusing on histopathologic cellular findings in fine-needle aspiration<br><br>(D) RECIST criteria, primarily developed for monitoring solid tumors over time in imaging<br><br>(E) UICC staging, mainly used for classifying tumor stages in the overall oncologic context		Learning objective: knowledge of classification systems for thyroid tumors.<br><br>Specification: recall and state the appropriate classification system to use in the context of thyroid ultrasound.		25

6<br><br>NM	Which radiotracer is particularly suited to depict cerebral metabolism in neurodegenerative diseases such as Alzheimer's disease?<br><br>(A) Rubidium-82<br><br>(B) Fluorine-18 fluorodeoxyglucose (FDG)<br><br>(C) Yttrium-90 microspheres<br><br>(D) Gallium-68 PSMA<br><br>(E) Technetium-99m MIBI		Learning objective: knowledge of PET tracers used in nuclear medicine diagnostics (positron-emitting radiopharmaceuticals).<br><br>Specification: recall and state an appropriate tracer for brain PET in the diagnostic workup of dementia.		32
7<br><br>NM	Which statement best describes the mechanism of the tracer commonly used in SPECT for Parkinson's diagnostics?<br><br>(A) 99mTc-HMPAO distributes according to cerebral blood flow and is used to assess focal perfusion deficits.<br><br>(B) 18F-FDG accumulates in metabolically active brain regions and allows assessment of glucose utilization.		Learning objective: knowledge of tracers used for diagnosing Parkinson's disease with SPECT (single-photon emission computed tomography).<br><br>Specification: recall and state the correct tracer and its mechanism of action in Parkinson diagnostics.		14

	(C) <u>123I-ioflupane binds selectively to presynaptic dopamine transporters in the striatum and reflects their integrity.</u><br><br>(D) 68Ga-PSMA binds to prostate-specific membrane antigen and depicts pathological processes in prostate cancer.<br><br>(E) 99mTc-MIBI shows mitochondrial activity in myocardial cells and is suitable for cardiac perfusion analysis.				
8<br><br>NM	Which of the following combinations of PET tracer and uptake principle is correctly matched?<br><br>(A) Fluorine-18 FET – binding to β-amyloid plaques<br><br>(B) Fluorine-18 FDG – transport via dopamine transporters<br><br>(C) <u>Gallium-68 DOTATATE – affinity for somatostatin receptors</u><br><br>(D) Fluorine-18 PSMA – accumulation in myocardial glucose metabolism<br><br>(E) Rubidium-82 – uptake via amino acid transport mechanisms		Learning objective: knowledge of the uptake characteristics of key positron emission tomography (PET) tracers.<br><br>Specification: recall and state the correct pairings of selected tracers with their underlying uptake mechanisms.		45

1<br><br>RO	What are the key objectives of follow-up care after radiation therapy?<br><br>(A) Verifying that radiation therapy was performed in accordance with technical standards<br><br>(B) <u>Early detection of tumor recurrence and management of treatment-related adverse effects</u><br><br>(C) Collecting epidemiological data on the long-term effects of radiation exposure in the population<br><br>(D) Documenting the delivered radiation dose to meet statutory reporting requirements<br><br>(E) Coordinating billing procedures within oncology follow-up programs	practical course; lecture	Learning objective: understanding the tasks and objectives of oncologic follow-up after radiotherapy.<br><br>Specification: follow-up care: content, objectives, and relevance to patient care and quality assurance.		33

2<br><br>RO	What characterizes the role of the classical main pillars in oncologic therapy?<br><br>(A) Radiotherapy is typically used only as a palliative measure.<br><br>(B) Systemic therapies generally replace surgical removal of the tumor.<br><br>(C) The choice of therapy takes into account tumor biology, location, and the patients' general condition.<br><br>(D) Surgical procedures are the primary therapeutic approach for hematologic neoplasms.<br><br>(E) The combination of the main pillars is carried out regardless of prognostic assessment.	practical course	Learning objective: fundamentals of oncologic treatment concepts.<br><br>Specification: the three classical pillars of cancer therapy – surgery, radiotherapy, and systemic therapy.		11

3<br><br>RO	Which effect is typical of concurrent chemoradiotherapy?<br><br>(A) The chemotherapeutic agents increase the radiosensitivity of tumor tissue (radiosensitizing).<br><br>(B) The chemotherapeutic agents shorten the duration of radiotherapy (radioshortening).<br><br>(C) Radiation side effects are neutralized by chemotherapeutic agents (radiocaring).<br><br>(D) Normal tissue is selectively protected (radioselecting).<br><br>(E) The radiation dose can be doubled without adjustment (radiodoubling).	practical course	Learning objective: fundamentals of radiation oncology – chemoradiation and combination strategies.<br><br>Specification: understanding the radiosensitizing effect of concurrent chemoradiotherapy.		27

4<br><br>RO	Which correspondence between dosimetric quantity and unit is correct?<br><br>(A) Organ dose – unit: sievert (Sv)<br><br>(B) Absorbed dose – unit: gray (Gy)<br><br>(C) Equivalent dose – unit: becquerel (Bq)<br><br>(D) Activity – unit: gray (Gy)<br><br>(E) Effective dose – unit: coulomb per kilogram (C/kg)	practical course; lecture	Learning objective: radiation protection – physical foundations of dosimetry.<br><br>Specification: knowledge of dose quantities and units used in radiology.		13
5	Which statement most accurately describes the radiobiological significance of a specific cell-cycle phase?<br><br>(A) Cells in S phase are particularly radiosensitive because DNA replication destabilizes the DNA structure.<br><br>(B) Cells in G1 phase exhibit increased radiosensitivity due to limited activity of DNA repair enzymes.<br><br>(C) Cells in mitosis are particularly radiosensitive because repair mechanisms are scarcely active during this phase.	lecture	Learning objective: radiation biology – cell-cycle dependence of radiosensitivity.<br><br>Specification: radiosensitivity varies across cell-cycle phases with respect to ionizing-radiation–induced DNA damage.		8

	(D) Cells in G0 phase are particularly radiosensitive because they do not divide for long periods.<br><br>(E) Cells in G2 phase are relatively radioresistant because they are preparing for cell division and activate protective mechanisms.				
6<br><br>RO	Why can treatment interruptions in curative radiotherapy negatively affect local tumor control?<br><br>(A) A longer overall treatment time reduces the technical precision of radiation delivery.<br><br>(B) Tumor cells die more during interruptions due to recruitment of immune cells.<br><br>(C) Over longer treatment courses, tumor cells may better adapt to sublethal radiation damage.<br><br>(D) <u>Interruptions can promote tumor cell repopulation between fractions.</u><br><br>(E) Interruptions automatically reduce the total dose.	practical course	Learning objective: impact of radiotherapy parameters on tumor control.<br><br>Specification: the role of total treatment time and unplanned interruptions in curative radiotherapy.		41

7<br><br>RO	What is the purpose of thermoplastic masks in head-and-neck radiotherapy?<br><br>(A) To evenly distribute the delivered radiation dose within the tumor tissue.<br><br>(B) To control the radiation source during fraction delivery.<br><br>(C) To reduce the radiation dose to adjacent organs by shielding scatter radiation.<br><br>(D) <u>To ensure precise immobilization and reproducibility of patient positioning at each fraction.</u><br><br>(E) To reduce the total delivered dose through mechanical compression.	practical course	Learning objective: fundamentals of radiotherapy – patient positioning and treatment planning.<br><br>Specification: function and purpose of thermoplastic masks for immobilization and treatment field setup.		16

8<br><br>RO	Which late effect can occur after curative radiotherapy for tumors of the genitourinary tract?<br><br>(A) Acute radiation pneumonitis with cough and shortness of breath<br><br>(B) Neuropathies of the upper extremities<br><br>(C) Fibrosis of the bladder or rectal tissue with functional impairment<br><br>(D) Radiation-induced cataract with progressive worsening of vision<br><br>(E) Hyperthyroidism as a consequence of endocrine radiation effects	practical course; lecture	Learning objective: recognize and contextualize long-term effects of radiotherapy in malignant disease.<br><br>Specification: radiotherapy for genitourinary tumors – adverse and late effects.		19

S 2 → Expert Item Review Instrument (English)

Purpose: To obtain blinded expert ratings on (i) curricular appropriateness, (ii) didactic quality, and (iii) perceived item origin for each multiple-choice question (MCQ).

Raters and blinding: Four experts (blinded to item origin) rated their assigned items independently.

Response format: For each item, complete the following fields:

1) Appropriateness of the question
Task: Please rate whether the item is suitable for use in an undergraduate medical examination in the relevant specialty (Radiology, Nuclear Medicine, or Radiation Oncology). (Target group: medical students; not intended for physicians in specialist training.)
Response format: Select a value 1–7 from the drop-down menu (1 = not appropriate; 7 = highly appropriate).

2) Didactic quality of the question
Task: Please rate the didactic quality. Consider, in particular, the presence/absence of hidden cues, the length and clarity of the stem and answer options, and the sensible use of negatively worded items (e.g., "… is not true").
Response format: Select a value 1–7 from the drop-down menu (1 = very poor; 7 = excellent).

3) Perceived item origin
Task: In your judgment, was this item authored by a human or generated by ChatGPT (LLM)?
Response format: Select "Human" or "ChatGPT" from the drop-down menu.

4) Comments (optional)
Task: If you wish to justify your ratings or add any remarks about the item (e.g., ambiguity, factual concerns, phrasing suggestions), please enter free text here.

Item list used for expert review (identifiers only)

Items are referenced by ID (01–48), Item Code (A = human; B = LLM), and specialty (radiation oncology (RO), radiology (RA), nuclear medicine (NM)). Full item stems and answer keys are provided in Supplementary Information S1. (Note: Item origin codes were masked to raters during review.)

ID	Item Code	Specialty
03	A	RO
05	A	NM
09	A	RA
11	B	RO
12	B	RA
14	B	NM
15	A	RO
18	A	RA
19	B	RO
22	A	RA
23	A	NM
24	B	RA
25	B	NM
27	B	RO
28	A	RO
30	A	RO

33	B	RO
35	B	NM
36	A	RA
38	B	RA
43	B	RA
44	A	NM
45	B	NM
47	A	NM
RO = radiation oncology, RA = radiology, NM = nuclear medicine.		
